# Supplementary material for: Mouse models of hereditary hemochromatosis do not develop early liver fibrosis in response to a high fat diet
Source: PLoS One. 2019 Aug 23;14(8):e0221455. doi: 10.1371/journal.pone.0221455 (PMC6707558; doi:10.1371/journal.pone.0221455)
Supplement: Attachment — Submitted filename: Rebuttal.docx [file pone.0221455.s001.docx]

**Response to Reviewer’s Comments**

The main concern of both reviewers was the considerable discrepancy in iron content of the standard diet and the high fat diet. In response to this fully justified criticism, we decided to redesign the study and perform an additional experiment using iron-matched standard and high fat diets. Other specific comments are addressed below.

**Reviewer 1**

1) *All the results in this study are based on animal experiments using regular and high fat diet (Harlad Tekland 2018 and Harlad Teklad TD.88137, respectively). The formula of each diet and the concentration of iron should be mentioned in the manuscript. The big difference (4 times) of iron concentration between standard diet and high fat diet (p 11, line 17) made further interpretation more complexed and difficult as pointed out by the authors.*

The revised manuscript contains links to the datasheets of the Harlad Tekland 2018 and Harlad Teklad TD.88137 diets. In addition, we explain that the custom-made TD.180571 high fat diet was identical to TD.88137, except that it was supplemented by the provider with extra iron to match the 200 ppm iron content of the standard Harlad Teklad 2018 diet.

2) *Figure 1 shows the effect of HFD on body weight and iron parameters such as serum iron, serum ferritin, transferrin saturation, and TIBC (transferrin). In this condition, both transferrin saturation (iron transport) and serum ferritin (iron storage) were decreased in HFD groups. In order to explain the mechanism, at least the iron content of two diets should be equalized; otherwise it is very difficult to make further interpretation.*

Fig. 1 in the revised manuscript shows additional data with iron-matched HFD. The new experiments were performed by the two new co-authors of the manuscript since the first author is no longer working in the lab. Because of a misunderstanding, they did not register body weights of the mice at the endpoint, and did not obtain serum ferritin values. We decided to keep body weight data of mice on standard and TD.88137 diets because this shows the weight gain in response to HFD intake. We also decided to omit the old serum ferritin data for clarity.

3) *Figure 2 shows the biochemical parameters such as glucose, triglyceride, cholesterol, HDL-cholesterol, ALT, and AST. Glucose is increased and TG is decreased in all groups, while cholesterol and HDL-cholesterol are increased except Hfe-/- group. The results are described in the text, but few interpretations were drawn about this interesting phenomenon. Cholesterol and TG (fatty acids) are categorized lipid as whole, but the anabolic and catabolic pathways are completely different. There should be any speculation or interpretation in the text, especially on the effect of Hfe.*

We agree that the relatively smaller (yet statistically significant) increases in cholesterol and HDL-cholesterol that were observed in HFD-fed Hfe-deficient mice is interesting. However, we noted that in the new experiment with iron-matched HFD, cholesterol and HDL-cholesterol values were increased similarly in all genotypes. We suspect that the differences may be due to experimental variations and, therefore, we avoided speculation in the text.

4) *The explanation of liver iron in Fig 4 is reasonable. However, considering the effect of lipid on iron metabolism and fibrosis in hemochromatosis model, at least the experimental model should show the increased iron in the tissue even in the high fat burden.*

This critical issue is now addressed with the new experiment using iron-matched diets.

5) *The results of Masson’s trichrome stain and a-SMA immune-stain are reasonable and encouraging for future development of fibrosis. The semi-quantification of the strength or area of positive is better to be shown.*

We have previously detected α-SMA immunoreactivity in livers of Hjv-/- mice that was not associated with histologically detectable fibrosis (references 13 and 15). Since the observation in Fig. 6 is not novel, we think that semi-quantification of the images will not add any new critical information.

6) *The striking difference of glycogen density an mitochondrial morphology in Hfe-/- , Hjv-/- and Hfe-/-Hjv-/-, suggesting the metabolic functions of Hfe and Hjv under EM, Biochemical or molecular confirmation is needed to find out the target of Hfe and Hjv. At least, further speculation or working hypothesis should be drawn in addition to the signaling pathway of iron-hepcidin regulatory pathway.*

We agree that the morphological differences are striking and understanding the mechanisms is important. We have previously reported the low glycogen density in Hjv-/- livers (ref. 13). We recently obtained proteomics data investigating this phenomenon, which we are currently analyzing. At this stage, we prefer to avoid speculation and just added the following sentence in the text: “Future experiments, for instance by using high-throughput -omics technologies, could shed more light on relevant biochemical mechanisms and pathophysiological implications”.

7) *(minor) The formula and iron content of regular and high fat diet should be described in the text or shown in the table*.

Please, see response to point 1).

8) (minor) *The title “mouse models of hereditary hemochromatosis do not develop early liver fibrosis in response to a high fat diet” does not seem to be appropriate. In this experimental mode, it seems unlikely to develop fibrosis even in the later stage. The title should be modified.*

We are not sure what will happen at a later stage. We discuss previous literature showing that chronic (at least 1 year) feeding of hemochromatosis mouse models with high iron diets does indeed lead to liver fibrosis (new references 24 and 25). Therefore, we prefer to keep the original title in the revised manuscript.

**Reviewer 2**

1) - *Since the standard and the HFD diets do not have a similar iron content (200 vs 50 ppm), data between controls and HFD groups are not comparable. Therefore, the authors should perform the study with matched diets for iron content.*

*- Since the Hfe-/-, Hjv-/- and Hfe-Hjv-/- mice have a similar liver iron content than WT mice on standard diet, they do not assess in this study the role of iron on NAFLD progression. This study should be repeated with a HFD containing an appropriated amount of iron.*

This major issue has been addressed with the new experiment using iron-matched HFD.

2) *Hepcidin has been shown to play a major role on fibrosis and obesity. Accordingly, the authors should present the hepcidin levels in their experiments and discuss them with regards of at least the 2 cited studies.*

We fully agree with this comment. We recently analyzed inflammatory hepcidin induction in the mouse models used herein and demonstrated that Hfe is dispensable, while Hjv is essential for this response (Fillebeen et al, Blood, 2018). Therefore, we expect that the absence of early liver fibrosis in our models is independent of the hepcidin pathway.
